# Tracing the origin of the crayfish plague pathogen, *Aphanomyces astaci*, to the Southeastern United States

**DOI:** 10.1038/s41598-021-88704-8

**Published:** 2021-04-29

**Authors:** Laura Martín-Torrijos, María Martínez-Ríos, Gloria Casabella-Herrero, Susan B. Adams, Colin R. Jackson, Javier Diéguez-Uribeondo

**Affiliations:** 1 Department of Mycology, Real Jardín Botánico-CSIC, Plaza Murillo 2, 28014 Madrid, Spain; 2 USDA Forest Service, Southern Research Station, Center for Bottomland Hardwoods Research, 1000 Front Street, Oxford, MS 38655 USA; 3 Department of Biology, University of Mississippi, University, MS 38677 USA

**Keywords:** Pathogens, Fungal genetics

## Abstract

The oomycete *Aphanomyces astaci* is an emerging infectious pathogen affecting freshwater crayfish worldwide and is responsible for one of the most severe wildlife pandemics ever reported. The pathogen has caused mass mortalities of freshwater crayfish species in Europe and Asia, and threatens other susceptible species in Madagascar, Oceania and South America. The pathogen naturally coexists with some North American crayfish species that are its chronic carriers. Presumptions that *A. astaci* originated in North America are based on disease outbreaks that followed translocations of North American crayfish and on the identification of the pathogen mainly in Europe. We studied *A. astaci* in the southeastern US, a center of freshwater crayfish diversity. In order to decipher the origin of the pathogen, we investigated (1) the distribution and haplotype diversity of *A. astaci*, and (2) whether there are crayfish species-specificities and/or geographical restrictions for *A. astaci* haplotypes. A total of 132 individuals, corresponding to 19 crayfish species and one shrimp species from 23 locations, tested positive for *A. astaci*. Mitochondrial rnnS and rnnL sequences indicated that *A. astaci* from the southeastern US exhibited the highest genetic diversity so far described for the pathogen (eight haplotypes, six of which we newly describe). Our findings that *A. astaci* is widely distributed and genetically diverse in the region supports the hypothesis that the pathogen originated in the southeastern US. In contrast to previous assumptions, however, the pathogen exhibited no clear species-specificity or geographical patterns.

## Introduction

During the past few decades, fungal and fungal-like pathogens have caused several worldwide pandemics responsible for declines in wildlife populations—even causing extinctions^1–5^. Globalization facilitates these pandemics—usually consequences of the transport and introduction of exotic and invasive species^6–8^. Moreover, habitat alterations due to anthropogenic activity break down natural dispersal barriers, allowing invasive species (frequently carrying pathogens)^9–15^ to further expand their ranges. Climate change alters environmental conditions, further benefitting some invasive species and favoring the development and spread of disease^2,4^.

Fungal and fungal-like pathogenic species have impacted freshwater ecosystems particularly strongly, causing a global decline in freshwater biodiversity that is far greater than that seen in terrestrial ecosystems^16,17^. For example, the panzootic chytrid fungus *Batrachochytrium dendrobatidis* originated in Asia and spread globally due to amphibian trade, causing declines in more than 500 amphibian species over the past half-century^18,19^. Furthermore, fungal-like pathogens, such as *Saprolegnia diclina* and *Saprolegnia ferax* (Oomycetes), are also responsible for mass extinctions in amphibians^20,21^ and may be spread by the fish trade^22^. Another pathogenic oomycete, *Aphanomyces invadans,* causes epizootic ulcerative syndrome (EUS), affecting more than 100 fish species in Asia, Australia, North America and Africa^23,24^.

Similarly, *Aphanomyces astaci* causes the crayfish plague in native European, Asian and Australian crayfish species^25–28^ and has decimated crayfish populations in those continents^5,6^. This oomycete is a specialized pathogen in freshwater crayfish^27,29,30^, one third of which are threatened with extinction globally^31^. The pathogen coexists naturally with North American crayfish but can efficiently colonize non-North American crayfish, almost without resistance^26^. In addition, *A. astaci* has spread rapidly throughout the world through translocations of North American chronic carriers^25,28–40^. In non-North American crayfish, crayfish plague infections typically cause death within a few days^41^.

**Figure 1:**
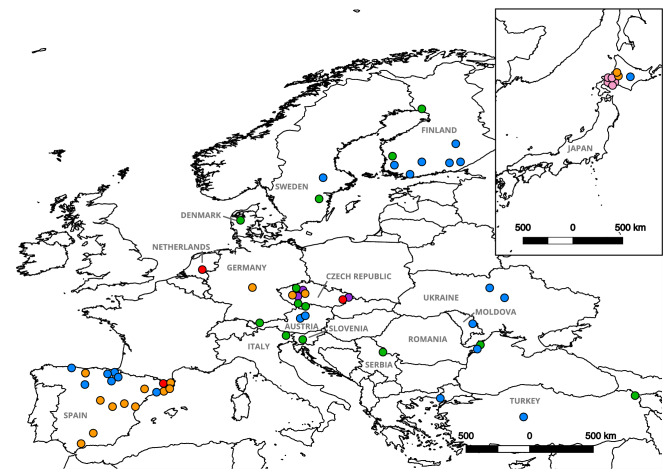
Distribution of *Aphanomyces astaci* haplotypes detected from locations in Europe and Japan^33,52,53^. Each point and color represent the presence of an *A. astaci* haplotype based on concatenated mitochondrial rnnS and rnnL regions. Colors indicate the haplotype code at each site as follows: green—a-haplotype, blue—b-haplotype, orange—d1-haplotype, red—d2-haplotype and pink—d3-haplotype [Maps were prepared using QGIS 2.14 (https://www.qgis.org/en/site/)].

Presumptions about the origin of the crayfish plague were based on disease outbreaks that followed historical translocations of North American crayfish species to many countries for aquaculture, sport fishing, or aquarium pet trade^42^. The first known introduction of a North American crayfish and subsequent crayfish plague outbreak was recorded in Europe in the nineteenth century^43^. Later, additional large-scale introductions of North American crayfish species were made in European and non-European countries^34–37,39,40,44–49^. Moreover, illegal translocations resulted in new crayfish plague outbreaks that decimated native crayfish populations in many countries^33,46,50^ (Fig. 1). Thus, *A. astaci* was listed among the 100 of the World’s Worst Invasive Alien Species^51^.

Knowledge about the virulence and genetic variability of *A. astaci* has come primarily from studies of crayfish plague outbreaks in Europe and Asia^50,52,54–60^. Specifically, mitochondrial DNA regions of *A. astaci* have been informative in assessing genetic diversity in both pure cultures and clinical samples of this clonally reproducing pathogen. To date, the mitochondrial DNA variability found in crayfish plague outbreaks in Europe and Japan has been allocated to six haplotypes (a, b, d1, d2, d3 and e-haplotypes) (Fig. 1) within two lineages^33,38,52,53,61^. However, only three studies have confirmed the presence of the pathogen in North America, revealing only two of the previously described haplotypes: a and b-haplotypes^62–64^.

Although evidence strongly supports a North American origin of *A. astaci*, our understanding of the crayfish plague pathogen in North America is still insufficient. A clearer understanding of the diversity and distribution of *A. astaci* within its native range is needed, not only to improve our comprehension of the evolution and epidemiology of pandemic pathogens, but also to determine future management and research directions. Similar questions have been faced when studying other emerging pathogens. For example, despite the occurrence of *Batrachochytrium dendrobatidis* in Asia, the lack of lethal outbreaks evidenced an endemic host–pathogen interaction in that region. Several studies have confirmed that the geographic origin of chytridiomycosis was in Asia, explaining the survival of Asian amphibian populations and stable host–pathogen dynamics^19^.

Although more than 428 crayfish species are native to North America^65^, the diversity, distribution and prevalence of *A. astaci* there is still largely unknown. Within North America, the southeastern US harbors the highest number of endemic crayfish species. The region represents not only a center of diversity, but also one of the two distinct origins of freshwater crayfish^6,52,66^. The presence of *A. astaci* has not been investigated in this crayfish-rich region even though such knowledge would improve our understanding of the origin and diversity of the pathogen. Thus, the main aim of this study was to evaluate the southeastern US as the possible center of origin of the crayfish plague pathogen *A. astaci.* For this purpose, we tested key questions including: (1) what is the distribution and haplotype diversity of *A. astaci* in the southeastern US, and (2) are *A. astaci* haplotypes crayfish species-specific and/or geographically restricted. In order to perform this study, we isolated and analyzed the pathogen from 30 distinct crayfish populations comprising a total of 21 crayfish species and one shrimp from five states in the southeastern US.

## Results

### *Aphanomyces astaci* detection

**Figure 2:**
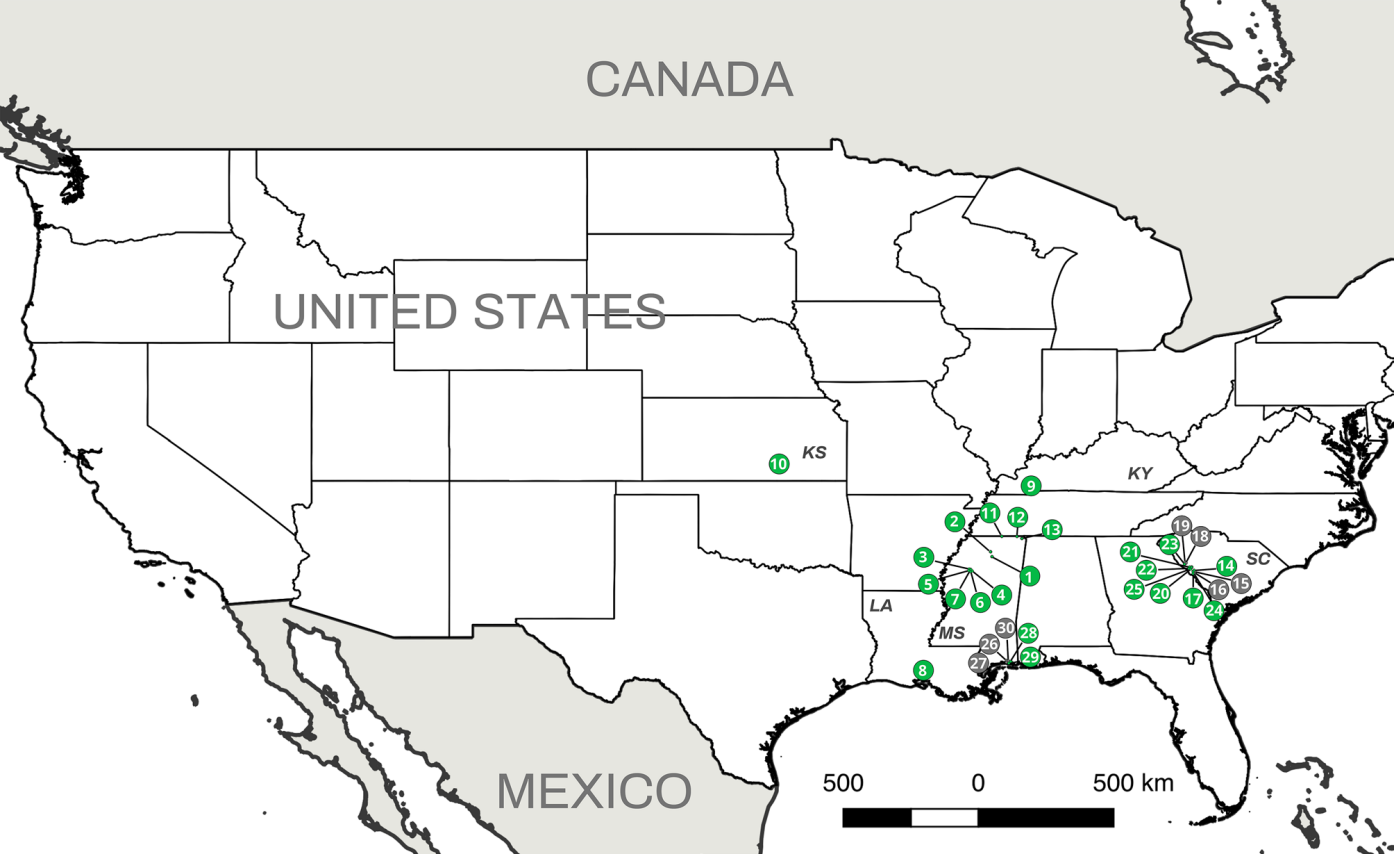
*Aphanomyces astaci* presence from the study locations. *Aphanomyces astaci* presence/absence is represented by green (positive) or grey (negative) circles. Numbers indicate the location code at each site (as in Supplementary Table 1). Two-letter state code is provided with the following abbreviation: KS (Kansas), KY (Kentucky), LA (Louisiana), MS (Mississippi) and SC (South Carolina). [Maps were prepared using QGIS 2.14 (https://www.qgis.org/en/site/)].

We obtained a total of 391 crayfish from 30 locations in five states (Kansas, Kentucky, Louisiana, Mississippi and South Carolina) (Fig. 2). The crayfish represented six genera and 21 species: *Cambarellus shufeldtii, Cambarus latimanus, Cambarus striatus, Cambarus tenebrosus, Creaserinus fodiens, Creaserinus oryktes, Faxonius etnieri* species complex*, Faxonius* sp., *Faxonius tricuspis, Faxonius wrighti, Lacunicambarus ludovicianus, Procambarus ablusus, Procambarus acutus, Procambarus clarkii, Procambarus hayi, Procambarus hybus, Procambarus pubescens, Procambarus raneyi, Procambarus troglodytes, Procambarus viaeviridis* and *Procambarus vioscai* (Supplementary Table 1). Additionally, one species of freshwater shrimp (*Palaemon kadiakensis*) was sampled and analyzed for the presence of *A. astaci.*

From 392 individuals, 132 crayfish and one shrimp tested positive for the *A. astaci* ITS region*,* 102 tested negative and 158 were not analyzed (i.e., the crayfish did not molt). *Aphanomyces astaci*-positive samples came from 23 locations and included 19 crayfish species: *C. shufeldtii, C. latimanus, C. striatus, C. fodiens, C. oryktes, F. etnieri* species complex*, Faxonius* sp.*, F. tricuspis, F. wrighti, P. ablusus, P. acutus, P. clarkii, P. hayi, P. hybus, P. pubescens, P. raneyi, P. troglodytes, P. viaeviridis* and *P. vioscai)* and one species of shrimp (*Palaemon kadiakensis*) (Fig. 2 and Supplementary Table 1). The ITS sequences (specific primers 42 and 640) for the 132 clinical samples were 99.82% identical to sequences of *A. astaci* available in GenBank (e.g., sequence FM999249-isolate SAP302) and identical to each other.

### Sequence analyses and haplotyping of *A. astaci*

**Figure 3:**
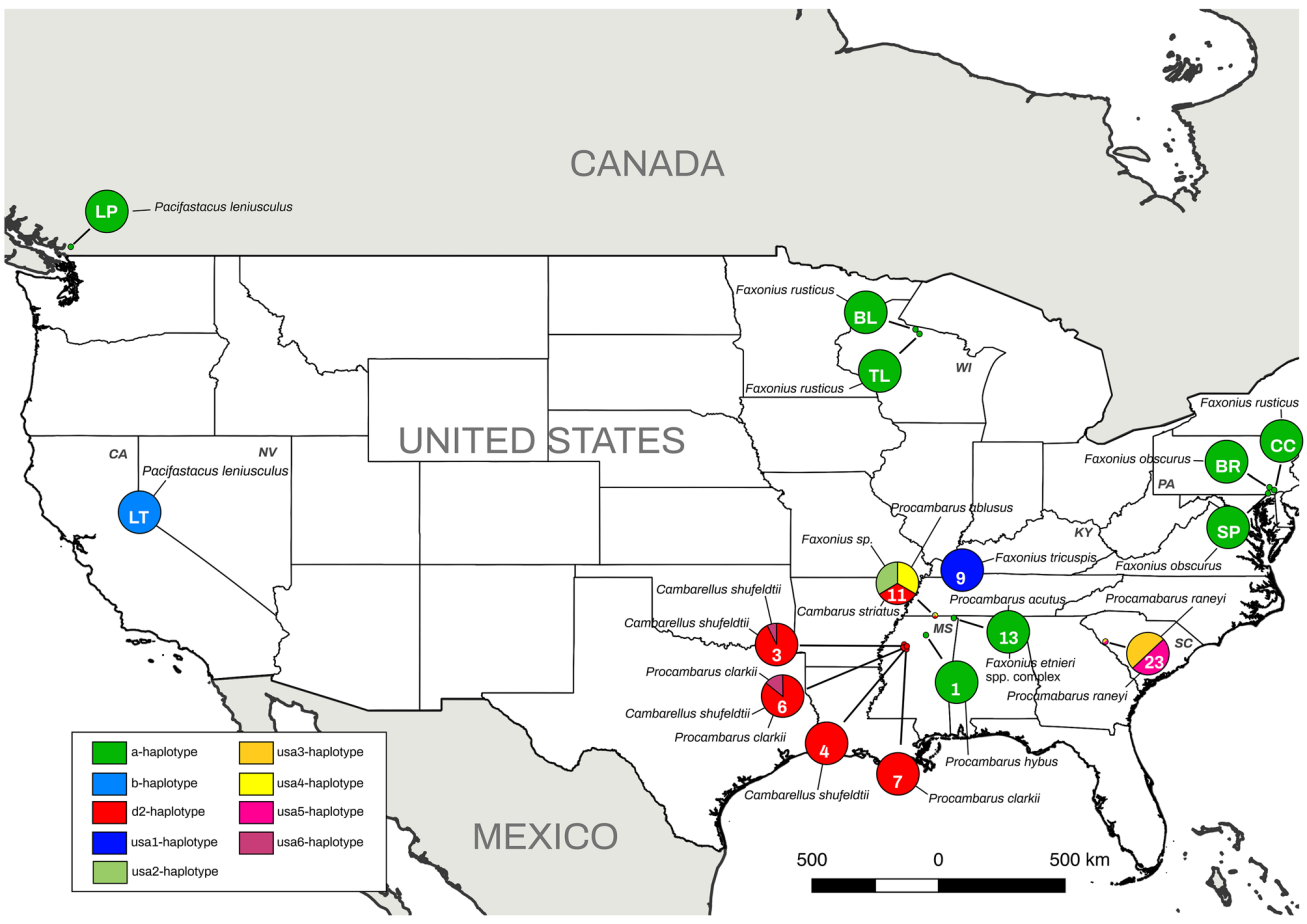
*Aphanomyces astaci* haplotypes detected from locations (numbers on pie graphs indicate locations; see Table 1) in North America. Haplotype frequencies indicated by relative proportion of the pie graph. Each color of the legend represents a different haplotype based on concatenated mitochondrial rnnS and rnnL regions. Crayfish species hosting different haplotypes are indicated at each location (also in Table 1). Location code LP corresponds to Lake Pitt^50^, LT to Lake Tahoe^63^, BL to Big Lake^62^, TL to Trout Lake^62^, CC to Chickies Creek^64^, BR to Brubaker Run^64^ and SP to Sunfish Pond^64^. Two-letter State code is provided with the following abbreviation: CA (California) KY (Kentucky), MS (Mississipi), PA (Pennsylvania), NV (Nevada), SC (South Carolina) and WI (Wisconsin). [Maps were prepared using QGIS 2.14 (https://www.qgis.org/en/site/)].

Twenty crayfish clinical samples (taken directly from crayfish) and 12 pure cultures from nine locations and three states (Kentucky, Mississippi and South Carolina) (Fig. 3) contained enough of the pathogen DNA for amplifying both mitochondrial regions (i.e., clinical samples often harbor low pathogen DNA concentration).

**Figure 4:**
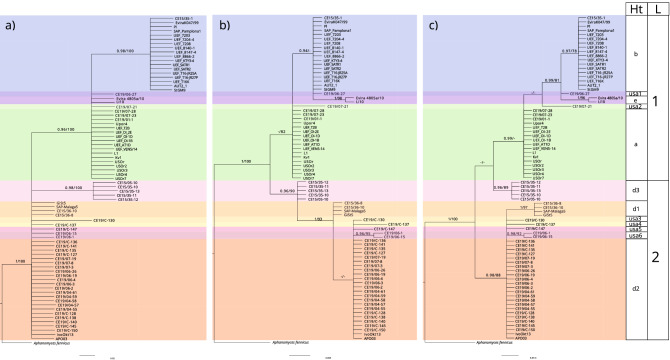
Phylogenetic analyses of *Aphanomyces astaci* mitochondrial regions. Bayesian phylogenetic analyses of *A. astaci* mitochondrial rnnS, rnnL and concatenated rnnS + rnnL sequences obtained from infected crayfish specimens and previous studies^33,52,53,62^. (**a**) Bayesian phylogenetic tree based on the rnnS sequences, (**b**) Bayesian phylogenetic tree based on the rnnL sequences, (**c**) Bayesian phylogenetic tree based on the concatenated rnnS + rnnL sequences. Values above the branches represent the Bayesian posterior probabilities (> 0.95) and ML bootstrap support values (> 75), respectively. Scale bar for phylogenetic analysis indicates substitutions per site. Abbreviations: Ht, haplotypes; L, lineages.

**Figure 5:**
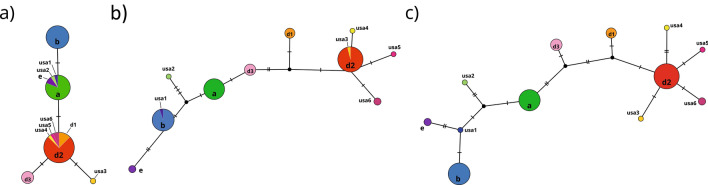
Haplotype network based on rnnS, rnnL and concatenated rnnS + rnnL mitochondrial DNA sequences, generated by statistical parsimony. The area of the circles is proportional to the number of sequences. (**a**) Haplotype network based on rnnS mtDNA sequences, (**b**) Haplotype network based on rnnL mtDNA sequences, (**c)** Haplotype network based on concatenated rnnS + rnnL mtDNA sequences. Mutation steps between haplotypes are shown as hatch marks.

For the phylogenetic approximations [Bayesian Inference (BI) and Maximum likelihood (ML)] and diversity estimations, we included a total of 78 sequences [32 sequences from the present study, 43 obtained from GenBank as reported from previous studies^33,52,62^ and two sequences from new *A. astaci* and *A. fennicus* isolates (CCRJB-75 and CCRJB-76, respectively)^67^ with 476 and 355 bp fragments of rnnS and rnnL amplicons, respectively. The phylogenetic approximations (BI and ML) supported the differentiation of the two lineages previously described^52^ (Fig. 4). The genetic diversity analysis confirmed and supported the phylogenetic analysis. Although both mitochondrial ribosomal rnnS and rnnL regions were informative, there were differences between them (Fig. 5). We obtained five haplotypes for the rnnS subunit (Fig. 5a), represented by four segregating sites (S), with a haplotype diversity (Hd) of 0.703, a nucleotide diversity (π) of 0.0022 and 1.018 average nucleotide differences (k). On the other hand, we obtained ten haplotypes for the rnnL subunit (Fig. 5b), represented by13 segregating sites (S), with a haplotype diversity (Hd) of 0.786, a nucleotide diversity (π) of 0.009 and 3.16 average nucleotide differences (k). However, concatenating rnnS and rnnL regions we confirmed a total of 12 haplotypes represented by 17 segregating sites (S), where 13 of them were parsimony informative (Fig. 5c). The concatenated sequences presented a haplotype diversity (Hd) of 0.801 with a nucleotide diversity (π) of 0.005 and 4.178 average nucleotide differences (k).

The phylogenetic approximations and the haplotype network confirmed the presence of eight haplotypes for the rrnS and rrnL concatenated regions among the analyzed samples: a, d2, usa1, usa2, usa3, usa4, usa5 and usa6-haplotypes (Figs. 4, 5) (Table 1). Six of these eight haplotypes (usa1, usa2, usa3, usa4, usa5 and usa6-haplotypes) are described and reported here for the first time, bringing the total number of known *A. astaci* haplotypes to 12. Moreover, these results were confirmed by an independent secondary molecular test (i.e., by repeating the amplification and sequencing of each of the haplotyped samples).

**Table 1:** Location and samples ID of the of the crayfish species analyzed for *Aphanomyces astaci* haplotypes.

Location code	Collection code	Host species	State	Sample type	DNA extraction	Species ID (rRNA)	Haplotype (rnnS/rnnL)	GenBank rnnS	GenBank rnnL
1	SA908-DEAD1	*Procambarus hybus*	Mississippi	Clinical sample	CE19/01-1	*Aphanomyces astaci*	*a*	MW346522	MW346542
3	SA927-1	*Cambarellus shufeldtii*	Mississippi	Clinical sample	CE19/06-12	*Aphanomyces astaci*	*usa6*	MW346516	MW346536
	SA927-5	*Cambarellus shufeldtii*	Mississippi	Clinical sample	CE19/04-58	*Aphanomyces astaci*	*d2*	MW346519	MW346539
	SA927-8	*Cambarellus shufeldtii*	Mississippi	Clinical sample	CE19/04-57	*Aphanomyces astaci*	*d2*	MW346520	MW346540
	SA927-16	*Cambarellus shufeldtii*	Mississippi	Clinical sample	CE19/06-2	*Aphanomyces astaci*	*d2*	MW346513	MW346533
	SA927-18*	*Cambarellus shufeldtii*	Mississippi	Clinical sample	CE19/06-26	*Aphanomyces astaci*	*d2*	MW346512	MW346532
	SA927-23	*Cambarellus shufeldtii*	Mississippi	Clinical sample	CE19/06-3	Aphanomyces astaci	d2	MW346510	MW346530
	SA927-25	*Cambarellus shufeldtii*	Mississippi	Clinical sample	CE19/04-55	*Aphanomyces astaci*	*d2*	MW346521	MW346541
	SA927-27	*Cambarellus shufeldtii*	Mississippi	Clinical sample	CE19/04-59	*Aphanomyces astaci*	*d2*	MW346518	MW346538
	SA927-18*	*Cambarellus shufeldtii*	Mississippi	Culture	CE19/C-136	*Aphanomyces astaci*	*d2*	MW346494	MW346481
	SA927-18*	*Cambarellus shufeldtii*	Mississippi	Culture	CE19/C-135	*Aphanomyces astaci*	*d2*	MW346495	MW346482
	SA927-18*	*Cambarellus shufeldtii*	Mississippi	Culture	CE19/C-127	*Aphanomyces astaci*	*d2*	MW346498	MW346485
	SA927-18*	*Cambarellus shufeldtii*	Mississippi	Culture	CE19/C-138	*Aphanomyces astaci*	*d2*	MW346492	MW346479
	SA927-14	*Cambarellus shufeldtii*	Mississippi	Culture	CE19/C-140	*Aphanomyces astaci*	*d2*	MW346491	MW346478
	SA927-18*	*Cambarellus shufeldtii*	Mississippi	Culture	CE19/C-150	*Aphanomyces astaci*	*d2*	MW346487	MW346474
4	SA928-7	*Cambarellus shufeldtii*	Mississippi	Clinical sample	CE19/06-4	*Aphanomyces astaci*	*d2*	MW346509	MW346529
6	SA930-2	*Cambarellus shufeldtii*	Mississippi	Clinical sample	CE19/04-61	*Aphanomyces astaci*	*d2*	MW346517	MW346537
	SA930-4	*Cambarellus shufeldtii*	Mississippi	Clinical sample	CE19/07-03	*Aphanomyces astaci*	*d2*	MW346504	MW346524
	SA930-6	*Procambarus clarkii*	Mississippi	Clinical sample	CE19/06-15	*Aphanomyces astaci*	*usa6*	MW346515	MW346535
	SA930-8*	*Procambarus clarkii*	Mississippi	Clinical sample	CE19/07-08	*Aphanomyces astaci*	*d2*	MW346503	MW346523
	SA930-8*	*Procambarus clarkii*	Mississippi	Culture	CE19/C-141	*Aphanomyces astaci*	*d2*	MW346490	MW346477
	SA930-8*	*Procambarus clarkii*	Mississippi	Culture	CE19/C-128	*Aphanomyces astaci*	*d2*	MW346497	MW346484
	SA930-8 *	*Procambarus clarkii*	Mississippi	Culture	CE19/C-145	*Aphanomyces astaci*	*d2*	MW346489	MW346476
7	M306-3	*Procambarus clarkii*	Mississippi	Clinical sample	CE19/06-19	*Aphanomyces astaci*	*d2*	MW346514	MW346534
9	SA937-5	*Faxonius tricuspis*	Kentucky	Clinical sample	CE19/06-27	*Aphanomyces astaci*	*usa1*	MW346511	MW346531
11	SA946-9	*Procambarus ablusus*	Mississippi	Clinical sample	CE19/07-19	*Aphanomyces astaci*	*d2*	MW346508	MW346528
	SA946-23	*Cambarus striatus*	Mississippi	Clinical sample	CE19/07-21	*Aphanomyces astaci*	*usa2*	MW346507	MW346527
	SA946-FAX SP JUV	*Faxonius sp.*	Mississippi	Culture	CE19/C-137	*Aphanomyces astaci*	*usa4*	MW346493	MW346480
13	SA948-1	*Procambarus acutus*	Mississippi	Clinical sample	CE19/07-23	*Aphanomyces astaci*	*a*	MW346506	MW346526
	SA948-5	*Faxonius etnieri*	Mississippi	Clinical sample	CE19/07-28	*Aphanomyces astaci*	*a*	MW346505	MW346525
23	SC32 MOLT 4*	*Procambarus raneyi*	South Carolina	Culture	CE19/C-130	*Aphanomyces astaci*	*usa3*	MW346496	MW346483
	SC32 MOLT 5*	*Procambarus raneyi*	South Carolina	Culture	CE19/C-147	*Aphanomyces astaci*	*usa5*	MW346488	MW346475

North American crayfish species from each of the analyzed locations (Location code:1–30), including the corresponding collection codes, state, sample type (clinical sample/culture), DNA isolation code (CE19/), BLAST species ID, result of the mitochondrial rnnS/rnnL haplotype and GenBank references for the rnnS and rnnL regions. NA: not applicable. Each unique collection code refers to an individual crayfish. *Different pieces from the same molt.

### Crayfish species, *A. astaci* haplotype diversity and distribution

The haplotyping results for the 20 crayfish clinical samples included five haplotypes: usa1-haplotype (one *Faxonius tricuspis*), usa2-haplotype (one *Cambarus striatus*), a-haplotype (one *Procambarus hybus*, one *Procambarus acutus* and one *Faxonius etnieri*), d2-haplotype (ten *Cambarellus shufeldtii*, two *Procambarus clarkii* and one *Procambarus ablusus*) and usa6-haplotype (one *Cambarellus shufeldtii* and one *Procambarus clarkii*) (GenBank accession numbers MW346503-MW346522 for rnnS and MW346523-MW346542 for rnnL) (Table 1). The amount of infection in the remaining samples that tested positive for *A. astaci* (ITS region) was too low to obtain conclusive results for both rrnS and rrnL.

Additionally, the haplotyping results for the 12 pure cultures included four haplotypes: usa3-haplotype (one *Procambarus raneyi* culture), usa4-haplotype (one *Faxonius* sp. culture), usa5-haplotype (one *Procambarus raneyi* culture) and d2-haplotype (six *Cambarellus shufeldtii* and three *Procambarus clarkii* cultures) (GenBank accession number MW346487-MW346498 for rnnS and MW346474-MW346485 for rnnL).

We found five scenarios relative to the distribution of *A. astaci* haplotypes among crayfish species at various locations: (1) one haplotype from one crayfish species (Locations 1, 4, 7 and 9), (2) two haplotypes from one crayfish species (Locations 3 and 23) (see below), (3) one haplotype from two crayfish species (Location 13), (4) two haplotypes from two crayfish species (Location 6) (see below), and (5) three haplotypes, one from each of three crayfish species (Location 11). At Location 23, the two haplotypes were recovered from one *P. raneyi* molt (i.e., we isolated the pathogen in two different pure cultures) and at Location 6, one haplotype was recovered from both species, and the second haplotype from only one of the species (Fig. 3) (Table 1).

## Discussion

We report and describe for the first time the presence, distribution and genetic diversity of the crayfish plague pathogen, *A. astaci*, in its potential center of origin, the southeastern US^68^. Previous studies regarding the origin, diversity, and distribution of *A. astaci* have addressed different questions. In the current study, we have explored several of them, including (1) the distribution and diversity of *A. astaci* in the southeastern US, and (2) whether *A. astaci* haplotypes are crayfish species-specific^50,54,55^ and/or restricted to a narrow geographic region of the pathogen’s native range^52,62,63^. Our results indicated that *A. astaci* is present and widely distributed in the southeastern US (e.g., it was present in 21 out of 23 sampling sites across the nine river basins we investigated) and possesses the highest genetic diversity of *A. astaci* described from any region to date. Previously, only two haplotypes (a and b-haplotypes) had been found in the five North American crayfish species examined from California, Michigan and Pennsylvania (*Pacifastacus leniusculus*, *Cambarus bartonii, Faxonius obscurus, Faxonius rusticus* and *Faxonius virilis*)^62–64^. However, in the southeastern US, we examined 21 North American crayfish species and found a total of eight haplotypes, six of which are previously unreported. This represents almost 70% of the *A. astaci* haplotype diversity known globally. The genetic diversity we describe is comparable to that of other pathogenic oomycetes. For example, in the US, 13 *Phytopthtora infestans* haplotypes were recently described using five mitochondrial loci^69^. Moreover, we showed that *A. astaci* can chronically colonize 19 additional North American crayfish species and also one species of freshwater shrimp (*Palaemon kadiakensis*). Previously, the only known report of a wild shrimp carrying *A. astaci* was of a *Macrobrachium lanchesteri* population from Indonesia that co-occurred with *P. clarkii*^70^. Only two (*Cambarus tenebrosus* and *Lacunicambarus ludovicianus*) of the 21 crayfish species that we sampled did not test positive for *A. astaci.* Thus, we confirm the presence of *A. astaci* in a wider distribution (Fig. 2) than previously described in North America^62–64^, confirming the origin of this pathogenic disease in North America.

Although the presence of the crayfish plague in North America was previously reported^62–64^, those studies examined a limited number of crayfish species and found haplotypes that had been previously isolated in Europe and Japan (i.e., a or b-haplotypes)^33,62–64^. Likewise, we confirmed the presence in North America of two haplotypes (a- and d2-haplotypes) first described in Europe. In the case of the a-haplotype, it was described as the first haplotype introduced to Europe in the nineteenth century^43,52^, yet no North American carrier has been described in the literature for this introduction. Two recent studies described *F. rusticus* and *F. obscurus* as the only North American crayfish known to host the a-haplotype^62,64^. We expanded the known range of this haplotype to include the southeastern US and added three additional native taxa as hosts: the *F. etnieri* species complex, *P. acutus* and *P. hybus*. In the case of the d2-haplotype, the only two North American crayfish described as carriers so far in Europe are *P. clarkii* and *Procambarus fallax virginalis.* As we expected, our study shows for the first time the presence of *A. astaci* in native populations of *P. clarkii*^54^, which carried the d2-haplotype and the newly discovered usa6-haplotype.

We confirmed that at least some *A. astaci* haplotypes are neither host species-specific nor narrowly distributed. Broadening and deepening our knowledge of *A. astaci* haplotype diversity and distribution, we recovered the d2-haplotype from two species in different genera (*Cambarellus* and *Procambarus*) in one river basin (Locations 3, 4, 6 and 7) and from a third genus (*Cambarus*) in a different river basin (Location 11) (Table 1). Although the a-haplotype was previously thought to be restricted to *F. rusticus* and *F. obscurus*^62,64^, we found it not only in another congener, but also in another genus (*Procambarus*). In addition, we documented the a-haplotype in two river basins in the southeastern US, even though it was previously thought to be restricted to the northern US. We also documented the d2-haplotype from two river basins. Our results indicated that *A. astaci* haplotypes tend to be neither host species-specific nor restricted to small geographic areas.

We also found instances of multiple haplotypes of *A. astaci* occurring in one species, in one individual, and in one location. We isolated two pure cultures of two different, but closely related, *A. astaci* haplotypes (usa3 and usa5) from a single individual (SC32 MOLT in Location 23). Also, multiple *A. astaci* haplotypes often co-occurred in a location. Within Location 11, we found three haplotypes from two lineages: usa2-haplotype from Lineage 1 and d2-haplotype and usa4-haplotype from Lineage 2. We also recovered two closely related haplotypes (d2 and usa6) from Locations 3 and 6 and two others (usa3 and usa5) from Location 23. None of the phenomena described have been documented previously. Our analysis showed no clear haplotype distributional patterns with respect to crayfish host species or geography. Thus, the biogeographic distribution of *A. astaci* genetic diversity within North America needs further investigation.

The pathogen’s genetic diversity and observed lack of species-specificity may have implications within North America as well as on continents where *A. astaci* is introduced. Crayfish have been frequently translocated within North America. For example, although *P. clarkii* and *F. virilis* are native in parts of North America, both are invasive beyond their native range, including west of the Great Divide where all native crayfish belong to the family Astacidae^71,72^. The genetic diversity of *A. astaci* suggests potential for intracontinental impacts from translocations of haplotypes. Presumably all North American crayfish are resistant to all haplotypes of *A. astaci,* considering that no mass mortalities have been observed after crayfish translocations. However, more subtle effects of translocated haplotypes with differing virulence from native haplotypes would not likely have been detected. Further understanding of the geographical distribution of *A. astaci* genetic diversity, its virulence, and the immune responses of crayfish to novel haplotypes would be beneficial for managing crayfish translocations and conserving native species in North America.

Our new approach of obtaining *A. astaci* from crayfish molts produced larger amounts of the pathogen for identification and isolation in pure cultures. This resulted in detection of *A. astaci* in 56.17% of the analyzed clinical samples. Instead of analyzing parts of the crayfish most susceptible to infection (i.e., soft abdominal cuticle, telson or walking legs)^73^, we waited until the molting period. Thus, we avoided the unnecessary killing of crayfish and maximized the amount of pathogen grown within the original host. Moreover, by incubating crayfish molts in distilled water for three days, we allowed the pathogen to continue growing both within and outside of the cuticle. By controlling the incubation temperature at 4 °C, we reduced bacterial blooms during the first stages of the pathogen isolation. Bacteria commonly surround the *A. astaci* hyphae, and antibiotics are often added to the PGA medium to inhibit bacterial growth. However, because bacteria rapidly develop resistance to these antibiotics, their addition becomes less effective over time. By reducing the incubation temperature and introducing a physical barrier^74^, we controlled bacterial blooms^75^ and obtained many clean isolates of the pathogen.

By using this new approach, we more readily detected and isolated *A. astaci*. Moreover, we strongly recommend isolating the pathogen in an axenic culture to assure an optimal concentration of the pathogen for analysis. In this study, we combined the sequencing of the ITS region (for identification of the *Aphanomyces* species)^76^ and the mtDNA (for identification of the *A. astaci* haplotype)^52^ in both clinical samples and pure cultures. Several studies have examined the genetic diversity of *A. astaci* using diverse methodologies (i.e., RAPD-PCR, the chitinase gene, AFLP, microsatellites)^50,52,56,58–60,77^. Although the mtDNA approximation^52^ requires a large concentration of the pathogen, it provides reliable results^67^ and enables detection of new diversity within *A. astaci* that might go undetected with other approximations^59,60^. Other approaches, such as eDNA monitoring^78^, could also be used to detect the presence of *A. astaci* in water samples and potentially to detect additional genetic diversity.

The uniquely high diversity of *A. astaci* haplotypes found in this study are an important step toward confirming the host–pathogen co-evolution between *A. astaci* and North American crayfish species. Our results suggest that further sampling in North America will reveal additional undiscovered *A. astaci* haplotype diversity vital to answering new host–pathogen co-evolutionary questions. Further, long-term monitoring might reveal emerging *A. astaci* diversity, depending on the rate of pathogen evolution.

## Methods

### Sample collection

We sampled 25 locations in five states between February and May 2019. Additionally, we included nine crayfish samples previously collected from five more locations and preserved in 95% ethanol. The specimens from Kansas were from an introduced population of *Procambarus clarkii* (Supplementary Table 1). Crayfish were captured by kick-seining and trapping and then held, separated by species and collection location, in the laboratory (US Forest Service, Southern Research Station, Center for Bottomland Hardwoods Research, in Oxford [Mississippi, USA]) until processed. Crayfish were kept individually in labeled, individual, plastic containers with chlorine-free water, aerators, gravel and medium size rocks. Each crayfish was kept alive until it molted and was subsequently preserved in 95% ethanol.

### Microscopic examination and *Aphanomyces astaci* isolation

For the microscopic examination, the molts were carefully removed and handled individually due to the fragility of the samples. Molts were kept in individual petri dishes with distilled water at 4 °C in order to reduce bacterial growth and optimize the growth of potential mycelium^75^. After three days, each molt was examined with an inverted microscope to check for the presence of growing hyphae. Each sample was divided into two parts: one for molecular identification and one for the pathogen isolation.

Pieces of the molt intended for molecular identification were transferred into 1.5-ml tubes and were frozen at − 80 °C until the DNA extraction was carried out in the laboratory at the Department of Biology at the University of Mississippi (UM). Samples were subsequently homogenized by manual mechanical disruption. A DNeasy Blood & Tissue Kit (Qiagen, Valencia, California, USA) was used to isolate genomic DNA.

Pieces of the molt intended for culture isolation were grown in Peptone Glucose Agar (PGA) at 4 °C. A selected agar plug was cut out from the resulting mycelia and inserted within an aluminum ring placed on a new PGA plate to protect the growing isolate from bacterial growth^74^. Plates were incubated at 4 °C for seven days, and each isolate was transferred into new PGA media once the hyphae spread under the metal ring. This process was repeated until no bacterial growth was observed using an inverted microscope. Additionally, a selected agar plug containing mycelia was placed in a 9 mm Petri dish containing 10 mL of liquid Peptone Glucose (PG-1) and incubated at room temperature for 48 h in order to obtain material for molecular identification. The obtained mycelium was transferred into 1.5-ml tubes and frozen at − 20 °C until the DNA extraction was carried out in the laboratory at UM. Samples were subsequently homogenized by manual mechanical disruption, and DNA extractions were carried out using an E.Z.N.A. Fungal DNA Mini Kit (Omega Biotek, Norcross, Atlanta, USA).

### *Aphanomyces astaci* detection and haplotyping

To test for the presence of the *A. astaci* pathogen, a fragment of the internal transcribed spacer (ITS) region was amplified using the diagnostic primers 42 (5′-GCTTGTGCTGAGGATGTTCT-3′)^73^ and 640 (5′-CTATCCGACTCCGCATTCTG-3′)^79^ (which amplify ITS1, the 5.8S rDNA and ITS2) in a single round of amplification according to the assay described by^73^. DNA extracted from a pure culture of *A. astaci* was used as the positive control; sterile Milli-Q water was used as the negative control. Amplified products (3 μL of each reaction) were analyzed by electrophoresis in 2% agarose SB gels stained with ethidium bromide and then purified using magnetic beads. Sequencing of both strands of positive products was performed using an automated sequencer (Applied Biosystems 3730xl DNA Analyzer, DNA Analysis Facility at Yale, USA and Applied Biosystems 3730xl DNA, Macrogen, The Netherlands). Sequences were aligned and edited using the program Geneious 10.0.2^80^. A BLAST search (NCBI database) was performed to verify the identity of each sequence.

Genomic DNA samples that tested positive for the presence of *A. astaci* with diagnostic primers 42^73^ and 640^79^ were used to characterize the phylogenetic relationships and haplotypes. The mitochondrial ribosomal small (rnnS) and large (rnnL) subunits were amplified using the primer pairs AphSSUF/AphSSUR (5′-AGCACTCCGCCTGAAGAGTA-3′ and 5′-GGGCGGTGTGTACAAAGTCT-3′) and AphLSUF/AphLSUR (5-AGGCGAAAGCTTACTATGATGG-3′ and 5′-CCAATTCTGTGCCACCTTCT-3′), respectively, as described by^52^. Positive and negative controls were included. Amplified products were analyzed by electrophoresis, purified, sequenced and aligned as described above.

Phylogenetic approximations based on Bayesian inference (BI) and maximum likelihood (ML) were used to reconstruct relationships. The BI analysis was performed in MrBayes v.3.2.6^81^ using the MCMC method with 10 million generations, three runs (8 chains per run) with a burn-in of 25% and a standard deviation of split frequencies < 0.01. Nodes with posterior probability (pp) values ≥ 0.95 were considered supported. The ML analysis was performed using RAxML v.8^82^, as implemented in raxmlGUI v1.5b1^83^, with 100 independent replicates and 1000 rapid bootstraps. Nodes with bootstrap values ≥ 75 were considered supported. The resulting trees from the BI and ML analyses were visualized with FigTree v1.4.2^84^. Sequences (57) corresponding to the mtDNA regions rnnS and rnnL of isolates analyzed in previous studies^33,52,62,67^, available in GenBank, were also included in our analyses. *Aphanomyces fennicus* was used as the outgroup in both phylogenetic approximations^67,76^. Analyses were performed with rnnS and rnnL individually, as well as with a concatenated rnnS and rnnL dataset, using the same parameters described above.

Genetic diversity was estimated by calculating the number of polymorphic (segregating) sites (S), the number of haplotypes, the haplotype diversity (Hd), the average number of nucleotide differences (k), and the nucleotide diversity (π) utilizing the program DNAsp v.5.10.01^85^. Mutational changes between sequences in the most parsimonious haplotype network were estimated using TCS v.1.21^86^, and the genealogical relationships were visualized with PopArt v1.7.2^87^.

### Ethics declarations

All experimental procedures and animal manipulations, as well as field sampling, were performed according to the US legislation.

## Supplementary Information

Supplementary Information 1.

Supplementary Information 2.

## Data Availability

All data generated or analyzed during this study are included in this published article (and its Supplementary Information files).
