# Supplementary material for: Tracing the origin of the crayfish plague pathogen, Aphanomyces astaci, to the Southeastern United States
Source: Sci Rep. 2021 Apr 29;11:9332. doi: 10.1038/s41598-021-88704-8 (PMC8085144; doi:10.1038/s41598-021-88704-8)
Supplement: Supplementary file 1 — Supplementary Information 1. [file 41598_2021_88704_MOESM1_ESM.docx]

**Supplementary Table 1:** Location and sample ID of the analyzed crayfish species. North American crayfish species from each of the analyzed locations (Location code:1-30), including the corresponding collection codes, date, locality, coordinates, molting state (YES/NO), DNA isolation code (CE19/), result of the amplification for *Aphanomyces astaci* specific primers 42F/640R (POSITIVE/NEGATIVE), BLAST species ID (rRNA), result of the mitochondrial rnnS/rnnL haplotype (Haplotype (rnnS/rnnL) and GenBank accession numbers for rnnS (GenBank rnnS) and rnnL (GenBank rnnL). NA: not applicable. NWR = National Wildlife Refuge.
